# Baseline characteristics of the HOMINY (human papillomavirus, human immunodeficiency virus, and oral microbiota interplay in Nigerian youth) prospective longitudinal cohort

**DOI:** 10.3389/froh.2026.1800454

**Published:** 2026-07-29

**Authors:** Erika Juhlin, Morgan C. Byrd, Rong Jiang, Toluwalope Gbolahan, Fidelis E. Eki-Udoko, Ozoemene N. Obuekwe, Yana Bromberg, Nicolas F. Schlecht, Modupe O. Coker, Nosayaba Osazuwa-Peters

**Affiliations:** 1 Department of Head and Neck Surgery & Communication Sciences, Duke University School of Medicine, Durham, NC, United States; 2 Duke Cancer Institute, Duke University, Durham, NC, United States; 3 Institute of Human Virology, Abuja, Federal Capital Territory, Nigeria; 4 Department of Child Health, University of Benin Teaching Hospital, Benin City, Nigeria; 5 Department of Oral and Maxillofacial Surgery, University of Benin, Benin City, Nigeria; 6 Department of Biology, Emory University, Atlanta, GA, United States; 7 Department of Computer Science, Emory University, Atlanta, GA, United States; 8 Department of Cancer Prevention and Control, Roswell Park Comprehensive Cancer Center, Buffalo, NY, United States; 9 Department of Oral Biology, Rutgers Biomedical and Health Sciences, Newark, NJ, United States; 10 Department of Biostatistics and Epidemiology, Rutgers School of Public Health, Piscataway, NJ, United States; 11 Department of Basic and Translational Sciences, Penn Dental Medicine, University of Pennsylvania, Philadelphia, PA, United States; 12 Department of Population Health Sciences, Duke University School of Medicine, Durham, NC, United States; 13 Duke Global Health Institute, Durham, NC, United States

**Keywords:** HPV vaccination, human immunodeficiency virus, human papillomavirus viruses, mother-child pairs, Nigeria, prospective cohort

## Abstract

**Background:**

The HOMINY (Human Papillomavirus, Human Immunodeficiency Virus, and Oral Microbiota Interplay in Nigerian Youth) cohort is a prospective longitudinal study established to investigate oral HPV infection and the oral microbiome among mother-child pairs with and without HIV in Nigeria. The objective of this analysis was to describe baseline demographic and clinical characteristics of the cohort, including HPV awareness, HPV vaccination, and cervical cancer screening practice by HIV status.

**Method:**

Mothers (aged ≥ 18) and children (aged 9–18) were prospectively enrolled at the University of Benin Teaching Hospital. Participants were grouped by HIV infection and exposure status. Baseline demographic, behavioral, and HPV-related data were collected using standardized questionnaires, medical record review, and case report forms. Group differences were assessed using parametric and non-parametric tests.

**Results:**

A total of 1,075 participants were enrolled. Among mothers, 68% were HIV-positive. Awareness of HPV (<7%) and the HPV vaccine (3.0%) was low with fewer than 1% having received HPV vaccination, and only 19.1% reported ever undergoing cervical cancer screening. Mothers living with HIV reported younger age at sexual debut, more lifetime sexual partners, and lower education attainment than HIV-uninfected mothers. Among children, awareness of HPV and the HPV vaccine was virtually absent, and only one child reported HPV vaccination. Birth characteristics differed by HIV status, with Caesarian sections and other perinatal exposures being higher among HIV-exposed groups.

**Conclusion:**

Baseline findings demonstrate substantial gaps in HPV awareness, vaccination, and cervical cancer screening among families affected by HIV before implementation of Nigeria's national HPV vaccination program. This highlights critical prevention gaps and sustained cancer risk, underscoring urgent needs for targeted HPV and HIV prevention in Nigerian youth. As the largest prospective mother-child cohort focused on oral HPV and HIV in sub-Saharan Africa, HOMINY provides an important foundation for future longitudinal studies of oral HPV infection, the oral microbiome, and the impact of HPV vaccination.

## Introduction

The HOMINY (Human Papillomavirus, Human Immunodeficiency Virus, and Oral Microbiota Interplay in Nigerian Youth) prospective cohort of mother-child pairs was established in 2022 as a longitudinal study to examine the natural history and complex interplay of the oral microbiome, oral human papillomavirus (HPV) infection, and human immunodeficiency virus (HIV) infection in Nigeria (1, 2). Nigeria has one of the world's largest HIV epidemics, with approximately 2 million people living with HIV, an estimated prevalence of 2.1% among adults ages 15–49 years (3). Persistent HPV infection remains an important public health concern because of its established role in cervical and other HPV-associated cancers (4). Women living with HIV are disproportionately affected by persistent HPV infection and HPV-associated malignancies (5–7), and cervical cancer remains one of the leading causes of cancer mortality among women in sub-Saharan Africa (8–10). Individuals living with HIV are also at increased risk for HPV acquisition and persistence (11), creating an important setting in which to examine the interaction between viral infections (12), host immunity, and the oral microbiome (13–15).

Beyond its microbiome objectives, the HOMINY cohort provides an opportunity to characterize baseline awareness of HPV, HPV vaccination, and cervical cancer screening among mothers and age-eligible children in Nigeria (16). HPV vaccination is the only established primary prevention for cervical, HPV-associated oropharyngeal cancer (17–20), and other HPV-associated malignancies (21). Public awareness of HPV and its prevention remain an essential component of successful vaccination programs as knowledge is consistently associated with vaccine acceptance and uptake. Prior studies from Nigeria have generally reported low HPV awareness and limited knowledge of HPV vaccination, with variation across regions and populations (22, 23). This gap is especially important in the context of Nigeria's 2023 national introduction of a free single-dose HPV vaccination for girls aged 9–14 years, which targeted approximately 7.7 million girls during its initial campaign, the largest single-round effort in Africa, starting with 16 states and the Federal Capital Territory (24, 25).

Baseline data collected before and during the early implementation of this national vaccination program provides an important opportunity to evaluate existing knowledge gaps, characterize populations that may benefit from future educational and prevention efforts, and identify barriers to HPV vaccination and cervical cancer screening uptake. The objective of this study was to describe baseline demographic and clinical characteristics of mother-child pairs enrolled in the HOMINY cohort and to evaluate HPV awareness, vaccination uptake, and cervical cancer screening practices according to HIV infection status. We hypothesized that participants living with HIV, who have more frequent contact with healthcare services, would demonstrate greater HPV awareness and higher utilization of preventive services than participants without HIV.

## Methods

### Study design and population

HOMINY was a prospective longitudinal study which examined the natural history of oral HPV infection in mothers living with or without HIV, and child participants who were either perinatally HIV- exposed or/and infected. The study was implemented at the University of Benin Teaching Hospital (UBTH, Benin City, Nigeria) as previously described (1, 2). Following written informed consent/assent (as appropriate), potential participants were screened for eligibility at baseline as follows: (i) adult mothers, 18 years or older, and (ii) children aged 9–18. Before recruiting paediatric participants, parental consent was sought, given the social and cultural sensitivity of sexually transmitted diseases in this community.

Baseline recruitment occurred between October 2022 and May 2023 at the UBTH general practice clinic, the paediatric special HIV clinic, and the dental clinics where individuals with HIV received specialized care. Each participant completed a baseline visit (visit 1) followed by three follow-up visits at approximately 6, 12, and 18 months after baseline. The longitudinal follow-up was designed to assess the natural history of oral HPV infection, characterize longitudinal changes in the oral microbiome, and evaluate how HIV infection and other factors influence HPV acquisition, persistence, and clearance over time. At each follow-up visit, participants underwent repeated questionnaire assessments and biological specimen collection according to the study protocol (1). This present analysis is restricted to baseline (visit 1) data to describe the cohort's initial demographic, behavioral, and HPV-related awareness and prevention characteristics prior to longitudinal follow-up.

### Ethics

The HOMINY study was approved by Institutional Review Boards at Rutgers State University (Pro2022000949), UBTH, Benin City (ADM/E22/A/VOL. VII/14813674), University of Pennsylvania (856699), and Duke University (Pro00112217). We obtained written informed consents from parents/guardians or caregivers, and assents (when appropriate for young children) before data collection.

### Data collection

Mothers were categorized as either living with HIV (HI) or HIV uninfected (HU). Children were classified into three groups based on both their own and their mother's HIV serostatus: HIV-infected (HI), HIV-perinatally exposed (i.e., has a mother living with HIV) but uninfected (HEU), and HIV-unexposed (i.e., has a mother living without HIV) and uninfected (HUU). Data on socio-demographics, behaviors, and knowledge of HPV and the HPV vaccine were collected using case report forms (CRFs) through interview-administered questionnaires and medical chart review. For child participants, pregnancy, birth, and early-life characteristics (e.g., maternal antiretroviral therapy (ART) during pregnancy, ART administration at birth, mode of delivery, membrane rupture, and preterm birth) were obtained from mothers or caregivers and supplemental by medical record review when available. Age-appropriate questions regarding HPV awareness, vaccination status, and behavioral characteristics were administered directly to child participants, with parental assistance provided when appropriate for younger children. The baseline visit CRF included additional information on type of delivery, whether antiretroviral therapy was received, and, if so, the timing and dosage of receipt. Completed CRFs were scanned and shared via an access-controlled folder and entered into Duke University's REDCap database (26). Data entry personnel noted missing data and discrepancies and queried the site study staff for resolution. Quality control measures included implementing data validation rules within REDCap and secondary layers of quality review. All identified issues were resolved.

At the baseline visit, mothers self-reported socio-demographic characteristics, including religion, marital history (ever married and current marital status), educational attainment (ever attended school and highest level completed), and employment status. Lifestyle behaviors were also assessed, including tobacco use (ever smoked or chewed) and alcohol use (ever and current use). HPV-related knowledge and behavior questions included current sexual activity, awareness of HPV and the HPV vaccine, receipt of at least one dose of the HPV vaccine, history of Pap smear screening and abnormal cervical screening results, sexual practices, history of laboratory-confirmed sexually transmitted infections (STIs) including STI status, age at sexual debut, number of lifetime sexual partners, number of pregnancies, and number of children.

### Data analysis

For the baseline visit, the prevalence of self-reported socio-demographic characteristics, behaviors, and HPV-related awareness was compared among HIV status groups for both mothers and children. Continuous variables were summarized using means and standard deviations (SD), and categorical variables using frequencies and percentages. Wilcoxon two-sample tests were performed for continuous variables and chi-square tests for categorical variables. Fisher's exact tests were used when any group had fewer than five participants. For child data, which included three comparison groups, *post-hoc* pairwise comparisons were conducted when overall tests were statistically significant to identify specific group differences. Missing data were reported but excluded from group comparisons. *P*-values were reported for each variable level. Statistical significance was defined as a two-sided *p* < 0.05. All analyses were conducted in SAS 9.4 (SAS Inc., Cary, NC). This report complies with the Strengthening the Reporting of Observational Studies in Epidemiology (STROBE) guidelines.

## Results

Baseline characteristics are presented by HIV status groups for mothers (Table 1) and children (Table 2). A total of 404 mothers were enrolled in HOMINY with 275 (68.1%) living with HIV. The mean age of mothers was 42.8 years (SD 5.4). Almost all participants were Christian (98.5%), 78.7% were currently married, and 98.3% had attended school, with over 25.3% completing at least a certificate or university education. Most mothers were employed as unskilled laborers (64.6%), while only 3.7% reported being unemployed. Overall, 37.4% reported ever consuming alcohol, of whom 51% reported current alcohol use, and less than 1% reported ever smoking or chewing tobacco. Awareness of HPV and the HPV vaccine was low, with only 7.4% and 3.0% of mothers reporting having heard of HPV and the HPV vaccine, respectively. Only two mothers (0.5%) reported receiving at least one dose of HPV vaccine. Approximately one-fifth of mothers (19.1%) had ever undergone a Pap smear, of whom 7.9% reported an abnormal result. Overall, 34.9% reported a history of laboratory-confirmed STI, and 25.2% reported a current laboratory-confirmed STI.

**Table 1:** Baseline characteristics of HOMINY (human papillomavirus, human immunodeficiency virus, and oral microbiota interplay in Nigerian youth) mother participants living with and without HIV.

Mother	Total		HI (infected)		HU (uninfected)		*p*
	*N* = 404		*N* = 275 (68.1%)		*N* = 129 (31.9%)		
Age (mean, SD)	42.8	5.4	43.2	5.1	42.1	6	0.1
Religion (*N*, %)							
Christian	398	98.5	271	98.5	127	98.4	1
Islam	6	1.5	4	1.5	2	1.6	
Current marital status							
Married	318	78.7	207	75.3	111	86.0	**0.02**
Separated	19	4.7	11	4.0	8	6.2	0.35
Divorced	3	0.7	3	1.1	0	0.0	0.55
Widowed	46	11.4	40	14.5	6	4.7	**0.003**
Unmarried	18	4.5	14	5.1	4	3.1	0.45
School level							
Primary	95	23.5	80	29.1	15	11.6	**<0.001**
JSS 3	40	9.9	29	10.5	11	8.5	0.53
SSS 3	155	38.4	103	37.5	52	40.3	0.58
Certificate	56	13.9	31	11.3	25	19.4	**0.03**
University	46	11.4	22	8.0	24	18.6	**0.002**
Other	5	1.2	3	1.1	2	1.6	0.66
No school	7	1.7	7	2.5	0	0.0	0.10
Employment							
Professional	35	8.7	15	5.5	20	15.5	**<0.001**
Clerical	45	11.1	20	7.3	25	19.4	**<0.001**
Skilled manual	46	11.4	35	12.7	11	8.5	0.22
Unskilled work	261	64.6	190	69.1	71	55.0	**0.006**
Unemployed	15	3.7	14	5.1	1	0.8	**0.04**
Other	2	0.5	1	0.4	1	0.8	0.54
Ever smoked tobacco?							
Yes	2	0.5	1	0.4	1	0.8	0.54
No	402	99.5	274	99.6	128	99.2	
Ever chewed tobacco?							
Yes	1	0.2	0	0.0	1	0.8	0.32
No	403	99.8	275	100.0	128	99.2	
Ever consume alcohol?							
Yes	151	37.4	107	38.9	44	34.1	0.33
No	251	62.1	166	60.4	85	65.9	
Missing	2	0.5	2	0.7	0	0.0	
Current alcohol consumption?							
Yes	77	19.1	48	17.5	29	22.5	**0.02**
No	74	18.3	59	21.5	15	11.6	
Missing	253	62.6	168	61.1	85	65.9	
Currently sexually active?							
Yes	336	83.2	228	82.9	108	83.7	0.84
No	68	16.8	47	17.1	21	16.3	
Have you heard of HPV?							
Yes	30	7.4	23	8.4	7	5.4	0.29
No	374	92.6	252	91.6	122	94.6	
Have you heard of HPV vaccine?							
Yes	12	3.0	7	2.5	5	3.9	0.46
No	392	97.0	268	97.5	124	96.1	
Have you ever received a dose of HPV vaccine?							
Yes	2	0.5	1	0.4	1	0.8	0.54
No	402	99.5	274	99.6	128	99.2	
Have you ever done a Pap smear?							
Yes	77	19.1	52	18.9	25	19.4	0.92
No	326	80.7	222	80.7	104	80.6	
Missing	1	0.2	1	0.4	0	0.0	
Abnormal cervical screening?							
Yes	6	1.5	3	1.1	3	2.3	0.38
No	69	17.1	48	17.5	21	16.3	
Missing	329	81.4	222	80.7	105	81.4	
Sex practices							
Oral yes	49	12.1	33	12.0	16	12.4	0.91
Oral no	355	87.9	242	88.0	113	87.6	
Vaginal yes	404	100.0	275	100.0	129	100.0	NA
Vaginal no	0	0.0	0	0.0	0	0.0	
Anal yes	20	5.0	15	5.5	5	3.9	0.50
Anal no	384	95.0	260	94.5	124	96.1	
History of a laboratory confirmed STI?							
Yes	141	34.9	90	32.7	49	38.0	0.29
No	263	65.1	184	66.9	79	61.2	
Missing	2	0.5	1	0.4	1	0.8	
Current laboratory confirmed STI?							
Yes	102	25.2	70	25.5	32	24.8	0.85
No	299	74.0	202	73.5	97	75.2	
Missing	3	0.7	3	1.1	0	0.0	
Sexual history							
At what age at first sexual experience (Mean, SD)?	19.6	3.8	19.0	3.6	20.8	4.0	**<0.0001**
Number of lifetime sexual partners (Mean, SD)?	3.5	3.3	3.9	3.7	2.8	2.2	**<0.0001**

Bold values indicates statistical significance at *p* < 0.05.

**Table 2:** Baseline characteristics of HOMINY (human papillomavirus, human immunodeficiency virus, and oral microbiota interplay in Nigerian youth) child participants among HIV status groups.

Child	Total		HUU (uninfected child uninfected mother)		HI (infected child infected mother)		HEU (uninfected child infected mother)		*p*
	*N* = 671		*N* = 226 (33.7%)		*N* = 225 (33.5%)		*N* = 220 (32.8%)		
Age (mean, SD)	12.5	2.4	12.7	2.5	12.7	2.5	12.2	2.3	**0.04**
Youth gender									
Male	326	48.6	111	49.1	110	48.9	105	47.7	0.96
Female	345	51.4	115	50.9	115	51.1	115	52.3	
School level									
Never been to school	1	0.1	0	0.0	1	0.4	0	0.0	0.66
Primary	244	36.4	69	30.5	94	41.8	81	36.8	**0.045**
JSS 3	249	37.1	85	37.6	75	33.3	89	40.5	0.3
SSS 3	166	24.7	66	29.2	53	23.6	47	21.4	0.14
Certificate	2	0.3	0	0.0	1	0.4	1	0.5	0.55
University	8	1.2	6	2.7	0	0.0	2	0.9	**0.02**
Missing	1	0.1	0	0.0	1	0.4	0	0.0	
Delivery									
Vaginal	518	77.2	167	73.9	184	81.8	167	75.9	0.12
C-section	77	11.5	25	11.1	13	5.8	39	17.7	**<0.001**
Assisted vaginal	46	6.9	19	8.4	21	9.3	6	2.7	**0.01**
Emergency C-section	30	4.5	15	6.6	7	3.1	8	3.6	0.15
How did the membrane rupture?									
Spontaneous	510	76.0	169	74.8	181	80.4	160	72.7	**0.04**
Induced/Artificial	150	22.4	54	23.9	37	16.4	59	26.8	
Missing	11	1.6	3	1.3	7	3.1	1	0.5	
Currently sexually active?									
Yes	6	0.9	1	0.4	5	2.2	0	0.0	0.11
No	5	0.7	0	0.0	2	0.9	3	1.4	
Missing	660	98.4	225	99.6	218	96.9	217	98.6	
History of a laboratory confirmed STI?									
Yes	1	0.1	0	0.0	1	0.4	0	0.0	0.66
No	667	99.4	226	100.0	221	98.2	220	100.0	
Missing	3	0.4	0	0.0	3	1.3	0	0.0	
Current laboratory confirmed STI?									
Yes	4	0.6	0	0.0	1	0.4	1	0.5	0.55
No	665	99.1	226	100.0	220	97.8	219	99.5	
Missing	2	0.3	0	0.0	4	1.8	0	0.0	
Have you had any sexual experience?									
Yes	11	1.6	1	0.4	7	3.1	3	1.4	0.07
No	659	98.2	225	99.6	217	96.4	217	98.6	
Missing	1	0.1	0	0.0	1	0.4	0	0.0	
Sex practices									
Oral yes	4	0.6	1	0.4	2	0.9	1	0.5	0.85
Oral no	667	99.4	225	99.6	223	99.1	219	99.5	
Vaginal yes	11	1.6	1	0.4	7	3.1	3	1.4	0.07
Vaginal no	660	98.4	225	99.6	218	96.9	217	98.6	
Anal Yes	2	0.3	1	0.4	1	0.4	0	0.0	1
Anal No	669	99.7	225	99.6	224	99.6	220	100.0	
Have you started your period?									
Yes	176	26.2	63	27.9	43	19.1	60	27.3	0.63
No	164	24.4	52	23.0	60	26.7	52	23.6	
Missing	331	49.3	111	49.1	112	49.8	108	49.1	
Was s/he born prematurely?									
Yes	20	3.0	2	0.9	7	3.1	11	5.0	**0.02**
No	651	97.0	224	99.1	218	96.9	209	95.0	
Have you heard of HPV?									
Yes	0	0.0	0	0.0	0	0.0	0	0.0	NA
No	670	99.9	226	100.0	225	100.0	219	99.5	
Missing	1	0.1	0	0.0	0	0.0	1	0.5	
Have you heard of HPV vaccine?									
Yes	0	0.0	0	0.0	0	0.0	0	0.0	NA
No	669	99.7	226	100.0	224	99.6	219	99.5	
Missing	2	0.3	0	0.0	1	0.4	1	0.5	
Have you ever received a dose of HPV vaccine?									
No	1	0.1	0	0.0	0	0.0	1	0.5	NA
Unknown	668	99.6	226	100.0	224	99.6	218	99.1	
Missing	2	0.3	0	0.0	1	0.4	1	0.5	

Bold values indicates statistical significance at *p* < 0.05.

Mothers living with HIV reported a greater of number of lifetime sexual partners (mean 3.9 [SD 3.7] vs. 2.8 [SD 2.2], *p* < 0.001) and a younger age at first sexual experience (19.0 [SD 3.6] vs. 20.8 [SD 4.0], *p* < 0.001) compared to HIV-uninfected mothers. Mothers living with HIV were less likely to be currently married (75.3% vs. 86.0%, *p* = 0.02) and more likely to be widowed (14.5% vs. 4.7%, *p* = 0.003). Educational attainment also differed significantly by HIV status. Mothers living with HIV were more likely to have completed only primary education (29.1% vs. 11.6%, *p* = 0.0001), and less likely to have completed a certificate (11.3% vs. 19.4%, *p* = 0.03) or university education (8.0% vs. 18.6%, *p* = 0.002). Employment patterns also differed, with mothers living with HIV being less likely to hold professional (5.5% vs. 15.5%, *p* = 0.0008) or clerical positions (7.3% vs. 19.4%, *p* = 0.0003) and were more likely to be employed as unskilled laborers (69.1% vs. 55.0%, *p* = 0.006) or be unemployed (5.1% vs. 0.8%, *p* = 0.04). Among mothers reporting alcohol use, current alcohol consumption was less common among mothers living with HIV than among HIV-uninfected mothers (17.5% vs. 22.5%, *p* = 0.02). No significant differences were observed by HIV status with respect to age, religion, tobacco use, current sexual activity, HPV awareness, HPV vaccination, Pap smear screening, abnormal cervical screening results, sexual practices, or history of laboratory-confirmed STIs (all *p* > 0.05).

A total of 671 children were enrolled in HOMINY, with approximately one-third represented in each HIV status group (HUU, HI, and HEU) and a balanced sex distribution. The overall mean age was 12.5 years (SD 2.4), children in the HEU group were significantly younger than those in both the HUU (12.2 vs. 12.7, *p* = 0.04) and the HI group (12.2 vs. 12.7, *p* = 0.02). Nearly all children had attended school, with only one child reported never having been enrolled. Educational attainment differed modestly by HIV status, with completion of primary school being less common among HEU than HI children (30.5% vs. 41.8%, *p* = 0.01), while no significant difference was observed between HEU and HUU groups.

Birth characteristics also differed across HIV status groups. Mode of delivery differed significantly overall (*p* < 0.001), with *post-hoc* analyses demonstrating differences among all three groups. Caesarean delivery was most common among HEU children (17.7%) and least common among HI children (5.8%). Assisted vaginal delivery was less common among HEU children than among HUU (2.7% vs. 8.4%, *p* = 0.01) and HI children (2.7% vs. 9.3%, *p* = 0.003). Spontaneous rupture of membranes was more frequently reported among HI children than in HUU children (80.4% vs. 72.7%, *p* = 0.01), while preterm birth was more common among HEU than HUU children (5.0% vs. 0.9%, *p* = 0.01). Sexual activity and laboratory-confirmed STIs were uncommon among children, with no significant differences across HIV status group. None of the children reported having heard of HPV or the HPV vaccine, and no child reported receiving an HPV vaccine.

## Discussion

The objective of this study was to describe the baseline characteristics of the HOMINY cohort, a prospective longitudinal study designed to investigate oral HPV infection and the oral microbiome among mother-child pairs with and without HIV. To our knowledge, with 1,075 participants, HOMINY is the largest prospective cohort of mother-child HIV dyads established to study oral HPV and the oral microbiome in sub-Saharan Africa. These baseline findings provide important context for future longitudinal analyses of oral HPV acquisition, persistence, clearance, and microbiome changes, while also providing a pre-implementation benchmark of HPV awareness, vaccination, and cervical cancer screening before Nigeria's national HPV vaccination campaign.

Although the primary focus of HOMINY is oral HPV, our findings also highlight important gaps in HPV-associated cancer prevention among women living with HIV. Most mothers were currently sexually active and reported multiple lifetime sexual partners, yet fewer than one in five reported ever having a Pap smear. These findings suggest missed opportunities for early detection of cervical precancer and cancer and are consistent with prior studies reporting low cervical cancer screening uptake across sub-Saharan Arica, particularly among women living with HIV. Financial barriers, limited access to screening services, low awareness, and sociocultural factors likely all contribute to poor screening uptake in the region. Although this population is considered vulnerable, participants face practical barriers to preventive care, including repeated clinic visits, HIV-related stigma, and other social challenges associated with long-term HIV care (27, 28).

We observed a substantial gap in HPV awareness and vaccination among mothers in the cohort. Only 7% of mothers had heard of HPV, 3% had heard of the HPV vaccine, and less than 1% reported ever receiving at least one dose of the vaccine. Although most mothers exceeded the age currently targeted for routine HPV vaccination in Nigeria (29), they remain the primary decision-makers for vaccine-eligible children. Their limited awareness of HPV therefore has clear implications for vaccine acceptance and uptake among adolescents. In the United States, adults aged 27–45 years may receive the HPV vaccine through shared clinical decision-making (30), a policy that has been associated with increased uptake in this age group (31). Importantly, these data were collected before the launch of Nigeria's first national single-dose HPV vaccination campaign on October 24, 2023 (25). The vaccine is provided free of charge by the Federal Ministry of Health through the National Primary Health Care Development Agency, with support from partners including Global Alliance for Vaccines and Immunization (Gavi), United Nations Children's Fund (UNICEF), and World Health Organization (WHO) (25). As such, these results provide a valuable baseline for evaluating future changes in awareness and vaccine uptake. As follow-up continues, HOMINY will help assess whether implementation of the national vaccination program translates into higher HPV awareness, greater vaccine uptake, and ultimately reduced oral HPV infection among children and adults.

The child cohort further underscores opportunities for improving HPV prevention. The mean age of enrolled children was 12.5 years, placing many within the age range targeted by Nigeria's national HPV vaccination program. Despite this, none of the children had heard of HPV or the HPV vaccine, and none of the 671 children reported receiving HPV vaccination. Approximately one-third of children in HOMINY were living with HIV, another one-third were HIV-exposed but uninfected, and nearly half were male. Current Nigerian guidelines provide free HPV vaccination only for girls aged 9–14 years (24), thereby excluding boys, including those living with HIV who remain at an elevated risk for HPV-associated malignancies (32, 33). Although cervical cancer prevention remains a primary objective of HPV vaccination programs in many low- and middle-income countries, increasing evidence demonstrates a growing burden of HPV-associated anal, penile, and oropharyngeal cancers among males (34–36). This burden may be further amplified among sexual minority populations, who, often due to cultural and society norms, experience significant stigma and discrimination that limit access to preventive healthcare services (37, 38). These findings support continued discussion of broader vaccination strategies that could reduce HPV-associated disease in high-risk populations.

Even among vaccine-eligible girls, additional challenges remain for children living with HIV. Current international recommendations advise that individuals with HIV or other immunocompromising conditions receive additional HPV vaccine doses to achieve optimal protection (17). However, Nigeria's national vaccination program currently provides a single-dose schedule, and additional doses must be purchased at a considerable cost (39). Combined with the limited employment opportunities reported by many mothers in our cohort and prior reports of unwillingness to pay for HPV vaccination in Nigeria (40), financial barriers may continue to limit completion of the recommended vaccination schedule among children living with HIV.

As HOMINY was specifically designed to investigate oral HPV, these baseline fundings also have implications beyond cervical cancer prevention. Oral HPV is the primary etiologic agent for HPV-associated oropharyngeal cancer, whose incidence continues to rise globally. Individuals living with HIV are at greater risk for persistent oral HPV infection and may therefore bear a disproportionate burden of HPV-associated oral disease. Improving HPV awareness and vaccination uptake in families affected by HIV could help reduce not only cervical cancer but also future oral HPV infection and HPV-associated oropharyngeal cancer. Continued follow-up of the HOMINY cohort will provide important insight into the natural history of oral HPV infection and the role of HIV-related immune dysregulation and the oral microbiome in HPV persistence and clearance.

Oral healthcare providers may also play an important role in HPV prevention. Dentists and other oral health professionals frequently encounter benign HPV-associated oral lesions and may help with early recognition of HPV-associated oropharyngeal disease (41). As trusted healthcare providers, they are well positioned to educate adolescents, parents, and caregivers regarding HPV transmission, oral HPV disease, and the benefits of HPV vaccination. Integrating HPV education into routine dental care may complement existing vaccination efforts and improve awareness in populations at increased risk of HPV-associated malignancies.

### Strengths and limitations

This study has several limitations. First, several variables, including HPV vaccination history, prior Pap Smear screening, sexual behaviors, alcohol use, tobacco use, and history of STIs, were self-reported and therefore may be subject to recall bias. In addition, because questions regarding sexual behaviors and substance use were collected in a culturally conservative setting, responses may have been influenced by social desirability bias, resulting in underreporting of sensitive behaviors. Although selected clinical information was supplemented by medical record review when available, some self-reported data could not be independently verified because of limited electronic health record availability. Finally, these analyses are limited to baseline data and therefore cannot yet evaluate longitudinal changes in HPV awareness, vaccination uptake, oral HPV infection, or oral microbiome composition.

Despite these limitations, HOMINY is, to our knowledge, the largest prospective mother-child cohort established to study oral HPV and HIV in sub-Saharan Africa. The baseline data provide an important pre-vaccination benchmark that will allow future evaluation of the impact of Nigeria's national HPV vaccination program on HPV awareness, vaccination uptake, and oral HPV epidemiology.

## Conclusion

Baseline data from the HOMINY cohort demonstrates extremely limited HPV awareness and HPV vaccine uptake among mothers and children before implementation of Nigeria's national HPV vaccination campaign. Given the elevated burden of persistent HPV infection and HPV-associated malignancies among individuals living with HIV, these findings identify substantial opportunities to improve HPV prevention, vaccination, and cancer screening in this vulnerable population. Continued longitudinal follow-up of the HOMINY cohort will provide important insight into the natural history of oral HPV infection and the influence of HIV and the oral microbiome on HPV persistence, while also evaluating the impact of natural HPV vaccination efforts in Nigeria.

## Data Availability

The datasets presented in this article are not readily available because the data may be made available upon reasonable request to the corresponding author. Requests to access the datasets should be directed to Nosayaba Osazuwa-Peters, nosa.peters@duke.edu.
